# A New Chapter for the Jornal Brasileiro de Pneumologia

**DOI:** 10.36416/3086-3910/e20260214

**Published:** 2026-05-22

**Authors:** Marcia Margaret Menezes Pizzichini

**Affiliations:** 1. Universidade Federal de Santa Catarina - UFSC - Florianópolis (SC) Brasil.; 2. Editora-Chefe do Respiratory Research & Clinical Practice, Brasília (DF) Brasil.

The Editors of the Jornal Brasileiro de Pneumologia (JBP) and the Board of Directors of the *Sociedade Brasileira de Pneumologia e Tisiologia* (SBPT, Brazilian Thoracic Society) have opted to rename the journal to enhance its international visibility, reach a broader community of respiratory physicians, and remove any geographic limitations associated with the original title. The new name, “Respiratory Research & Clinical Practice” (RRCP), reflects its broadened goals, vision, and mission. This strategic transition is not merely cosmetic; it represents a commitment to expanding our dialogue, fostering collaborative research, and addressing a diverse set of respiratory challenges globally.

As we embark on this journey, it is crucial to address the implications of this change. Over the next couple of years, the performance metrics of these journals will be calculated by averaging the statistics from JBP and the newly named RRCP. Consequently, while we anticipate a temporary decline in key performance indicators-including the Journal Citation Reports metrics such as the Impact Factor and our rankings within SCImago Journal & Country Rank-this should not be misinterpreted as a decline in quality or relevance. The RRCP is set to take its place on the international stage, and while we may experience some initial fluctuations in metrics during this transformative phase, it is essential to recognize that growth and excellence are often borne out of challenges and transitional periods.

Indeed, even as we prepare to transition, JBP has firmly established its reputation within the scientific community. The recent SCImago metrics^1^ report positions JBP favourably among 165 international respiratory journals, reflecting an improved score from 0.599 to 0.714, which has allowed us to maintain our status in the second quartile. Our ascent from 75th to 65th place among respiratory journals underscores our commitment not only to quality but also to the impact of the research we publish. With a current H-index of 55, we are solidifying our position as a leading voice in pulmonary health in Brazil and beyond.

Moreover, it is noteworthy that our standing among Brazilian medical journals has improved from 12th to 9th place, and we have been recognized with an upgrade from B1 to A2 in the Qualis Capes evaluation.^2^ These accolades not only highlight our robust contributions to the field of respiratory medicine but also reinforce our strategic position as we transition to RRCP.

This month marks a significant milestone as we present the second volume of RRCP, covering March-April 2026. To ensure clarity and continuity for our readers, RRCP retains the original numbering system of JBP; however, it now operates under a new ISSN. This move emphasizes that RRCP is not a new journal but rather an evolved platform for continuing our work with renewed enthusiasm and purposes.

In conclusion, we emphasize that the renaming of JBP to RRCP marks a positive and progressive step for our journal, reflecting our commitment to deeper engagement with the global respiratory community. Although we may experience some temporary fluctuations in our metrics, we are optimistic that this significant transition will establish a strong foundation for long-term growth and enhanced impact. We invite our community of researchers, clinicians, and mentors to join us in this exciting new chapter as we collaboratively advance the field of respiratory health worldwide.
